# Anion-Responsive Metallopolymer Hydrogels for Healthcare Applications

**DOI:** 10.1038/srep11914

**Published:** 2015-07-23

**Authors:** Jiuyang Zhang, Jing Yan, Parasmani Pageni, Yi Yan, Adam Wirth, Yun-Ping Chen, Yali Qiao, Qian Wang, Alan W. Decho, Chuanbing Tang

**Affiliations:** 1 Department of Chemistry and Biochemistry, University of South Carolina, Columbia, South Carolina 29208, USA; 2 Department of Environmental Health Sciences, University of South Carolina, Columbia, South Carolina 29208, USA

## Abstract

Metallopolymers combine a processable, versatile organic polymeric skeleton with functional metals, providing multiple functions and methodologies in materials science. Taking advantage of cationic cobaltocenium as the key building block, organogels could be simply switched to hydrogels via a highly efficient ion exchange. With the unique ionic complexion ability, cobaltocenium moieties provide a robust soft substrate for recycling antibiotics from water. The essential polyelectrolyte nature offers the metallopolymer hydrogels to kill multidrug resistant bacteria. The multifunctional characteristics of these hydrogels highlight the potential for metallopolymers in the field of healthcare and environmental treatment.

Hydrogels are water-swelling polymeric networks that could bear extraordinary performance in various aspects, such as good biocompatibility, tunable mechanical strength, and multi-stimuli response^1^,^2^,^3^,^4^. In the last few decades, various types of hydrogels have been developed, including, but not limited to, organic polymer-crosslinked^5^, protein-based, peptide-containing^6^,^7^, and supramolecular host-guest hydrogels^8^,^9^. By inserting functionalities in numerous matrix, hydrogels could be found applications for tissue engineering, drug delivery, matrix chemistry, artificial extracellular materials, to name just a few^4^,^5^,^10^,^11^.

Metallopolymers combine the synthetic efficiency, processability and versatility of an organic polymer framework with the unique redox, responsive and catalytic properties of inorganic metals, which have attracted great attention recently^12^,^13^,^14^,^15^,^16^. However, among the various known hydrogels, metallopolymer-based hydrogels are far less studied^9^,^17^,^18^,^19^,^20^. This may be attributed, in part, to concerns such as metal cytotoxicity and the difficulty to integrate metals into gel networks^19^,^21^,^22^,^23^. There are a few reports on metal ion-based hydrogels^18^,^19^,^20^,^24^, most of which have focused on chelating bonds^25^,^26^,^27^. As substitutes for metal ions, covalent-bonded (linked via covalent bonds) organometallic moieties are more desirable for hydrogels, which are more chemically stable, compared with non-covalent bonded counterparts. The combination of organometallics and polymer gels not only maintains attributes from polymeric networks, but also develops inherent functionalities from organometallic molecules as unique building blocks^20^,^26^,^28^,^29^,^30^. Metallocene-containing polymeric materials have been demonstrated for a variety of applications in the areas of biomedicines, energy storage, catalysts, templating precursors, and magnetic materials^13^,^31^,^32^,^33^,^34^,^35^,^36^. Given its signature redox chemistry, ferrocene has been used in hydrogels^9^,^17^,^29^, although the hydrophobicity and stability of ferrocene has limited its role mostly as an auxiliary building block for fabricating environmentally-compatible materials^33^,^36^. As 18-e analogies of ferrocene, cationic 18-e metallocenium ions (e.g. cobaltocenium) demonstrate several advantages over commonly applied ferrocene, including excellent hydrophilicity, high chemical stability and emerging bioactivities^23^,^37^,^38^,^39^.

Herein we report the first charged metallocenium-containing organogels and hydrogels, and their emerging applications in recycling antibiotics from water and in killing multidrug resistant bacteria (Fig. 1). Specifically, cationic cobaltocenium-containing polymeric networks exhibit some of most distinguished ion-responsive properties. With different counterions, charged cobaltocenium-containing gels exhibit drastically different physical behaviors, including mechanical properties and water uptake ability. Concurrently, these cationic gels are able to absorb diverse antibiotics from water. Taking advantage of the intrinsic antimicrobial properties of cobaltocenium polyelectrolytes, cobaltocenium-containing hydrogels can be further employed as novel antimicrobial biomaterials.

As shown in Fig. 2a, 2-cobaltocenium amidoethyl methacrylate hexafluorophosphate (CoAEMAPF_6_) was synthesized as a monomer with hexafluorophosphate (PF_6_ˉ) as the counterion (both ^1^H and ^13^C NMR spectra provided in Figures S1 and S2). Cationic cobaltocenium-containing organogels (PCoPF_6_-Gel) were subsequently prepared by free radical copolymerization of monomer CoAEMAPF_6_ and a crosslinker, poly(ethylene glycol) dimethacrylate (PEGDMA), in dimethylformamide (DMF). The formation of gel was partially dictated by the ratio of CoAEMAPF_6_ to PEGDMA. A free-standing gel could be prepared, when the molar fraction of PEGDMA was 7% (When more than 10% of the PEGDMA was used, the gel was very brittle and easy to crack in water, while with less than 5% PEGDMA, the crosslinking was not very effective and the resultant materials would be liquid-like instead of a solid gel.) On the other hand, the concentration of monomer also played a role in generating solid gels, as no gels were obtained at concentrations less than 0.66 g/mL.

In general, compositions of organic gels are usually difficult to be characterized due to the insolubility of their polymeric networks. In the case of PCoPF_6_-Gel, the compositions could be quantitatively determined using the characteristic UV-vis absorption of cobaltocenium moiety at ~270 nm (Figure S3A)^33^. After the gels were immersed in water for 3 days, the unreacted monomers could be completely removed. According to the standard calibration curve for monomer CoAEMAPF_6_ (Figure S3B), the concentration of unreacted monomer was calculated by UV-vis spectroscopy to be 44 μg/mL. The final purified and dried gel had a yield of 56.1%. Based on Equations 1 and 2, the conversion of monomers was 56%, in an excellent agreement with the yield. The weight fraction of cobaltocenium moieties in the gel was 67.3 wt%. The resultant organogel was brownish (Fig. 2a).


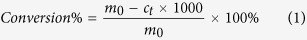



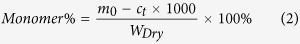


(In the equations, conversion represents the percentage of monomers reacted in final gels, *m*_*0*_is the amount of total monomers used before reaction; *C*_*t*_is the concentration of the diluted solution collected, which was obtained from the UV-vis absorption spectra and the monomer standard curve. *Monomer%* is the weight percentage of CoAEMAPF_6_ in final gels. *W*_*Dry*_ is the final weight of purified and dried PCoPF_6_-Gel)

According to recent work^34^,^40^, cobaltocenium-containing polymers are ion-responsive materials. With different counterions, their corresponding polymers show drastic difference in hydrophobicity. With counterions like PF_6_^−^ and tetraphenylborate (BPh_4_^−^), the cobaltocenium-containing polymers are relatively hydrophobic. In contrast, counterions, such as chloride, bromide and nitrate, result in polymers that are very hydrophilic. Thus we attempted to convert the organogel PCoPF_6_-Gel into a hydrogel by a recently-developed phase-transfer ion-exchange method (Fig. 2b)^34^. The anion exchange was done in acetonitrile with the aid of tetrabutylammonium chloride (TBACl), as shown in Scheme S2. All PF_6_^−^ anions in PCoPF_6_-Gel were replaced by Cl^−^ to form the hydrogel PCoCl-Gel. As shown by solid state ^31^P NMR (Fig. 2c), PCoPF_6_-Gel has a broad phosphorus peak at −144.4 ppm using sodium hexafluorophosphate as a reference with a peak at −149.3 ppm. After ion-exchange, no phosphorus peak was observed in PCoCl-Gel, indicating the complete removal of PF_6_^−^anions.

After ion-exchange, the light yellow PCoCl-Gel (Fig. 1) is hydrophilic that can have a water content as high as 96.4%, approximately 27 times of its dry gel weight. While for PCoPF_6_-Gel, the water content was 80% (only four times of the weight of dry gel), which probably also had a significant contribution from PEO crosslinkers due to the ability of hydrophilic PEO to retain water in gels. The structure of these two gels were also characterized by Field-Emission Scanning Electron Microscopy (FE-SEM). As shown in Figure S7, the chloride gel (PCoCl-Gel) was porous, indicating its potential to hold more water, while PCoPF_6_-Gel appeared much more compact. The drastic change in their morphologies, hydrophilicity and mechanical strength was mainly attributed to the unique ion-dependent solubilities of cobaltocenium-containing polymers. Furthermore, the mechanical properties of PCoCl-Gel and PCoPF_6_-Gel are significantly different. Figure. 3a illustrates the oscillatory strain results for both hydrogels. In comparison with PCoPF_6_-Gel, PCoCl-Gel exhibits a narrower linear viscoelastic region (LVR, about 0 ~ 25.8% changed to about 0 ~ 4.8%) and a lower critical strain (82.7% dropped to 10.7%), demonstrating a shortened elastic response range. A frequency sweep experiment was performed to compare the stiffness of both hydrogels (Fig. 3b). The storage modulus of both hydrogels was much higher than their loss modulus, and all of the values were relatively independent from frequency, which showed that both hydrogels behaved as a true gel over the frequency tested (range from 0.1 to 10 rad/s). Notably, PCoCl-Gel showed a weaker and more viscous gel nature than that of PCoPF_6_-Gel. Due to the same crosslinking degree for both gels, such difference in their mechanical properties was possibly resulted from the stronger electrostatic repulsive interactions in PCoCl-Gel than those in PCoPF_6_-Gel, which led to a looser network, as well as the increasing solubility of the more hydrophilic and ionic polymer networks in PCoCl-Gel, thus leading to lower stiffness.

Antibiotic-contamination is an urgent challenge in public health and environmental science, as it has deleteriously enhanced the evolution of bacterial resistance to commonly-used antibiotics such as β-lactam antibiotics^41^,^42^. Current techniques to remove β-lactam antibiotics are primarily based on oxidation methods (such as ozonation and fenton-oxidation)^43^,^44^, nanofiltration^45^,^46^, and photocatalytic reactions^46^,^47^. However, these techniques usually introduce new oxidizing chemicals into water and thus require special facilities^44^,^48^. On the other hand, adsorption (e.g. the use of activated carbons) is a more convenient and ‘clean’ strategy for removal of β-lactam antibiotics^48^,^49^,^50^. However, current adsorption materials are usually only suitable for antibiotics with high concentrations in the range of 50 mg/L to 200 mg/L. With some specific procedures (e.g. a combination of ozonation and ion-exchange columns), only a few of them could remove antibiotics with concentrations below 10 mg/L^50^,^51^,^52^. Recently we showed that cobaltocenium-containing polymers can form bioconjugates with β-lactam antibiotics (e.g. penicillin-G, amoxicillin, ampicillin and cefazolin) via ion-pairing between cationic metal centers and carboxylate anions^23^. Given the relatively strong ion-pairing capabilities of cobaltocenium, cationic PCoCl-Gel could be a promising novel material to remove antibiotics.

As shown in UV-vis spectra (Fig. 4c and Figure S4), after mixing dry PCoCl-Gel with different β-lactam antibiotics (amoxicillin, cefazolin and cefoxitin) in water (10 mg/L), ~75% of antibiotics were absorbed with a final concentration at ~2 mg/L (Fig. 4d). Compared with other adsorption techniques, such as ion-exchange columns and activated carbon with ~3 g/L absorbents for antibiotics at 50 ~ 200 mg/L in DI water (Resistivity: 18.2 MΩ × cm)^44^,^48^,^49^,^53^,^54^, PCoCl-Gel showed a better capacity to remove β-lactam antibiotics (1.5 g/L gel could efficiently treat with antibiotics waste at a concentration of 3 ~ 10 mg/L). After immersing PCoCl-Gel in the solution of antibiotics at a higher concentration (2 g/L), about 47% of the antibiotics were removed (Fig. 4a,b and S5), indicating that 1.0 mg PCoCl-Gel could load 0.42 mg antibiotics (based on theoretical calculation, see supporting information). Theoretically 1.0 mg PCoCl-Gel could absorb 0.60 ~ 0.80 mg antibiotics, assuming that the molar ratio of cobaltocenium to antibiotics is 1:1. The ability to remove antibiotics from aqueous solution by cobaltocenium moieties might be contributed by the ionic binding and coordination effects between cationic cobaltocenium and anionic antibiotics. Similar to techniques used in industry^44^,^48^,^49^,^53^,^54^, we performed an experiment in tap water (Resistivity: 5.7 Ω × cm) under the same conditions as above (4.5 mg gel in 3 mL tap water with 10 mg/L cefazolin antibiotics). The result showed that in the first 12 hours, the drug uptake could remove 44.5% antibiotics (Figure S6), which was lower than the one in DI water (~75%).

Cobaltocenium-containing methacrylate polymers have been reported to inhibit the growth of bacteria without any obvious toxicity toward human cells in *in vitro* and *in vivo* studies^23^. Cationic cobaltocenium-containing hydrogels may also have the similar ability to disrupt negative charged cell walls of bacteria. Thus we tested the antimicrobial activities of cobaltocenium-containing hydrogels against various bacteria, including drug-resistant strains. This unique property showed additional advantage over other kinds of absorbents for antibiotics. It could enable practical utilization of such hydrogels in the environment considering the existence of drug-resistant bacteria in antibiotics-contaminated water^41^,^42^. The antimicrobial efficacies of hydrogels are shown in Fig. 4e,f,g. The hydrogel PCoCl-Gel (at a concentration of 20 mg/mL) showed inhibition of the growth for Gram-negative *E-coli* (90% inhibition, Fig. 4e), Gram-positive *S. aureus* (80% inhibition; Fig. 4f), and hospital-acquired methicillin-resistant *S. aureus* (HA-MRSA, 80% inhibition, Fig. 4g)^23^,^55^. These data indicated that cobaltocenium-containing hydrogel can not only absorb antibiotics in contaminated water, but also inhibit the growth of drug-resistant bacteria in their environment. The antimicrobial ability of hydrogels originates from cationic cobaltocenium-containing macromolecules in the gels. Due to the much lower solubility of the gels compared to homopolymers, the concentration of inhibition increased about ~1000 times.

In conclusion, we report the preparation and application of novel ion-responsive metallopolymer gels. Using an ion-exchange technique, the gels showed a transition from organogels to hydrogels. In the presence of different counterions, these gels showed strikingly different mechanical, water-absorbing, and optical properties. Due to the ability of cobaltocenium moieties to bind β-lactam antibiotics, these hydrogels were additionally capable of absorbing antibiotics from contaminated water. Furthermore, these cationic hydrogels can not only efficiently remove antibiotics in water, but also inhibit the growth of different bacteria, including drug-resistant strains. As a class of novel polymeric gels, these cobaltocenium-containing hydrogels could open up new avenues for diverse applications, especially in the areas of biomedicines and environmental treatment.

## Methods

### Materials

2-Aminoethyl methacrylate hydrochloride (90%), *N*-(3-dimethylaminopropyl)-*N′*-ethylcarbodiimide hydrochloride (EDC-HCl, 98%), 4-(dimethylamino) pyridine and tetrabutylammonium chloride (TBACl) were purchased from Aldrich. Polyethylene glycol dimethacrylate (PEGDMA) (M.W. 3,400 g/mol) was purchased from VWR. *N,N-*Dimethylformamide (DMF) was dried and freshly distilled. Water was from Thermo Scientific Nanopure with ion conductivity at 18.2 MΩ. *Staphylococcus aureus* and *Escherichia coli* strains were purchased from ATCC: HA-MRSA (ATCCBAA-29213), and MSSA (ATCCBAA-1718), and *E-coli* (ATCC-25922). All other chemicals were from commercial sources and used as received.

### Characterization

^1^H NMR (400 MHz) spectra were recorded on a Varian Mercury 400 spectrometer with tetramethylsilane (TMS) as an internal reference. ^19^F NMR (376 MHz) spectra were recorded on a Varian Mercury 400 spectrometer with CHF_3_ as an internal reference standard. A 500 MHz Bruker Avance III-HD spectrometer with a microprocessor-controlled gradient unit was used for ^31^P Solid State NMR spectra. UV-vis spectra were recorded on a Shimadzu UV 2450 spectrophotometer. Mechanical measurement was performed on Discovery HR-3, hybrid rheometer (TA Instrument). The images of gels were recorded by Field-Emission Scanning Electron Microscopy (FE-SEM, Zeiss UltraPlus). The samples were firstly coated with gold using Denton Dest II Sputter Coater for 45 s and then observed by SEM. Gels were freeze-dried before using for SEM.

### Synthesis of 2-cobaltoceniumamidoethyl methacrylate hexafluorophosphate (CoAEMAPF_6_)

CoAEMAPF_6_ was synthesized based on an amidation reaction. Cobaltocenium carboxylic acid hexafluorophosphate (2 g, 5.29 mmol), 2-aminoethyl methacrylate hydrochloride (0.94 g, 5.68 mmol), and 4-(dimethylamino)pyridine (0.13 g, 1.06 mmol) were dissolved in 20 mL dichloromethane (DCM) and the solution was cooled to 0 ^o^C. EDC-HCl (1.1 g, 5.74 mmol) was slowly added into the above solution. Then, dry triethylamine (1.6 g, 15.8 mmol) was added into reaction. The reaction was stirred for 5 hours at room temperature. Then, solution was extracted by aqueous saturated sodium hexafluorophosphate solution three times to remove unreacted chemicals. The organic phase was collected, condensed and precipitated into diethyl ether. Yellow solids were collected and dried under vacuum overnight. Yield: 1.6 g, 58%. ^1^H NMR (Figure S1) (CD_3_COCD_3_, δ, ppm): 8.30 (broad, *NH*CH_2_, 1H), 6.42 (t, Cp, 2H), 6.22 (m, *CH*_*2*_C, 1H), 6.10 (t, Cp, 2H), 5.92 (s, Cp, 5H), 5.62 (m, *CH*_*2*_C, 1H), 4.42 (m, O*CH*_*2*_CH_2_NH, 2H), 3.66 (m, OCH_2_*CH*_*2*_NH, 2H), 1.94 (m, *CH*_*3*_CCO, 3H). ^13^C NMR (Figure S2) (CD_3_COCD_3_, δ, ppm): 138 (CH_2_*C*CO), 126 (*C*H_2_CCO), 83–86 (Cp ring), 62 (COO*C*H_2_), 39 (CH_2_*C*H_2_NH), 18 (*C*H_3_CCO). Mass spectrum: theoretical m/z: 344.07; found m/z: 344.00.

### Synthesis of cobaltocenium polymer gels (PCoPF_6_-Gel)

PCoPF_6_-Gel was synthesized via polymerization of PEGDMA and CoAEMAPF_6_. CoAEMAPF_6_ (0.3 g, 6.17 × 10^−1^  mol) and PEGDMA (145 mg, 4.26 × 10^−2^  mmol) were dissolved in 0.35 mL DMF in a test tube. AIBN solution (0.1 mL, 3 mg/mL, 1.83 × 10^−3^  mmol) was then added into the above solution. The solution was purged by nitrogen gas for 30 minutes and then placed in an oil bath under 90 ^o^C for 6 hours. The solid gel was then collected by immersing in 100 mL acetone for three days (change acetone every day) to remove unreacted monomers and crosslinkers.

### Characterization of PCoPF_6_-Gel compositions

The compositions of PCoPF_6_-Gel were characterized by measuring the UV-vis absorption of cobaltocenium moieties at ~270 nm. PCoPF_6_-Gel was prepared according to the above procedure using the following materials (0.1 g CoAEMAPF_6_, 48 mg PEGDMA, 0.1 mg AIBN and 0.15 mL DMF). After 6 hours, all materials in a test tube were collected and immersed in 15 mL nanopure (deionized) water for 24 h, and then the water was collected and another 15 mL nanopure water. This procedure was repeated in triplicate to remove unbound monomers from the gels, as shown in Scheme S1. In total, 45 mL of water solution was collected, then diluted to 50 mL. A 1 mL solution was then taken and diluted 20 times for UV-vis measurement. A standard procedure was repeated one more time. This procedure was aimed to remove monomers from the gels, as shown in Scheme S1. Totally 45 mL water solution was collected and diluted to 50 mL. 1 mL solution was then taken and diluted 20 times for UV-vis measurement. A standard curve (Figure S3B) for CoAEMAPF_6_ was established using a series of monomer solution (67 μg/mL, 50 μg/mL, 25 μg/mL, 12.5 μg/mL and 6.3 μg/mL). The compositions of the PCoPF_6_-Gel were calculated according to equations 1 and 2.

### Ion-exchange of PCoPF_6_-Gel

**Figure 2:**
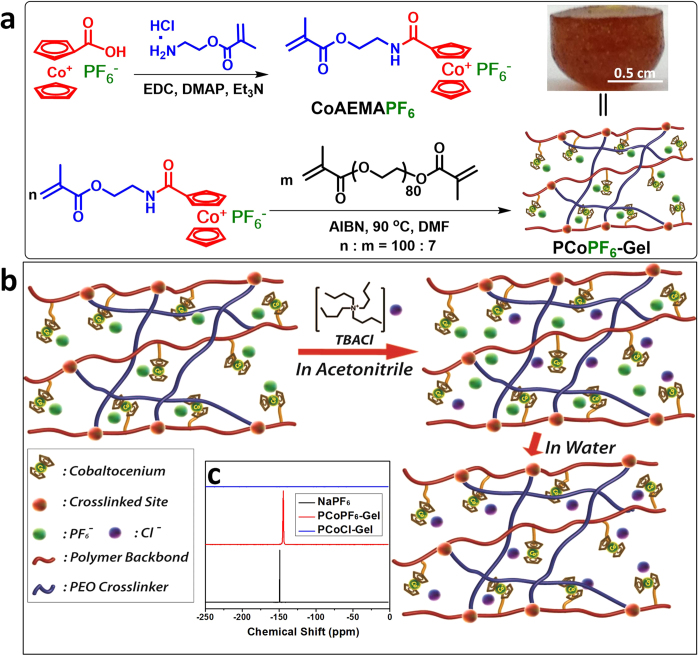
(**a**) Preparation of cationic cobaltocenium-containing organogels (PCoPF_6_-Gel) via copolymerization of CoAEMAPF_6_ and PEGDMA. (**b**) Preparation of chloride-paired hydrogel (PCoCl-Gel) from the organogel PCoPF_6_-Gel via ion-exchange with tetrabutylammonium chloride (TBACl). (**c**) Solid state ^31^P NMR spectra of organogel PCoPF_6_-Gel, hydrogel PCoCl-Gel and sodium hexafluorophosphate (NaPF_6_, as a reference).

Ion-exchange of the PCoPF_6_-Gel was performed according to Scheme S2. PCoPF_6_-Gel (0.1 g) was immersed in 20 mL acetonitrile with 0.1 g TBACl salt. After 24 hours, acetonitrile solvent was replaced by 20 mL fresh TBACl acetonitrile solution (0.1 g TBACl salt). The procedure was repeated one more time to ensure complete ion-exchange. After three ion-exchanges, the resultant gel was then immersed in 40 mL water for three successive times to remove acetonitrile and TBACl residuals. The swollen gel was then collected and dried for later use. The chloride-paired gel (PCoCl-Gel) was characterized by ^31^P Solid-State NMR (Fig. 2c) to ensure the complete removal of PF_6_ anions.

### Measurement of water uptake for PCoPF_6_-Gel and PCoCl-Gel

Water uptake by PCoPF_6_-Gel and PCoCl-Gel followed the same procedure. The weight of dried gels was first measured. After immersion in water for 24 hours, the weight of wet gels were then measured. The percent water uptake was calculated according to Equation 3 and data from Table S1:


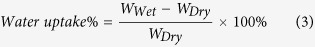


Equation 3,*W*_*Wet*_ means the weight of wet gel and *W*_*Dry*_ is the weight of dry gel.

### Antibiotic contaminant removal by cobaltocenium-containing hydrogels

**Figure 4:**
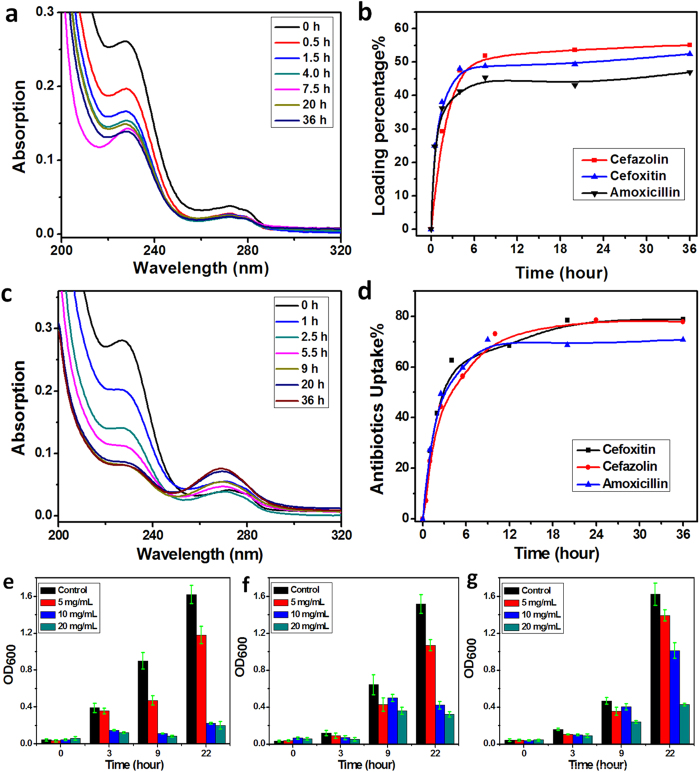
Treatment of antibiotics in water by cobaltocenium-containing hydrogels: PCoCl-Gel (dry weight: 4.5 mg) as an absorbent for β-lactam antibiotics: amoxicillin, cefazolin and cefoxitin; (**a**) UV-vis absorption curves of 2 mL, 2 g/L amoxicillin under different time intervals; (**b**) UV-vis absorption data for time-dependent antibiotics-uptake (2 mL, 2 g/L) of PCoCl-Gel; (**c**) UV-vis absorption curves of 10 mg/L amoxicillin under different time intervals; (**d**) UV-vis absorption data for time-dependent antibiotics-uptake under a low concentration (3 mL, 10 mg/L) of PCoCl-Gel. Inhibition of cobaltocenium-containing hydrogels (PCoCl-Gel) was observed against (**e**) Gram-negative *E-coli*; (**f**) Gram-positive *S. aureus*; and (**g**) HA-MRSA under different concentrations by standard solution micro-broth measurement.

Four antibiotic sodium salts, including penicillin-G, amoxicillin, ampicillin, cefazolin, were tested following the same procedure. PCoCl-Gel (dry weight = 4.5 mg) was immersed into 2 mL aliquots of aqueous antibiotic (2 mg/mL). At certain time intervals (0.0 h, 0.5 h, 1.5 h, 4.0 h, 7.5 h, 20.0 h, 36.0 h), 10 μL solution was removed and then diluted 20 times to measure UV-vis absorption of antibiotic in the solution at the following wavelengths: 230 nm for amoxicillin, 266 nm for ampicillin, and 230 nm for cefazolin. The concentration of antibiotic was obtained according to their respective standard curve profiles. The standard curves for each antibiotic were established by measuring UV-vis absorption for a series of solutions with different concentrations (1.56 μg/mL, 3.12 μg/mL, 6.3 μg/mL, 12.5 μg/mL, 25 μg/mL and 50 μg/mL). The removal of antibiotic at low concentrations (10 μg/mL) was also performed in DI water. PCoCl-Gel (dry weight = 4.5 mg) was immersed in 3 mL antibiotic aqueous solution (10 μg/mL). Samples were also taken at certain time intervals (0.0 h, 1.0 h, 2.5 h, 5.5 h, 9.0 h, 20.0 h, 36.0 h; see Fig. 4). The removal of antibiotic at low concentrations (10 μg/mL) was also performed in tap water (Resistivity: 5.7 Ω × cm).

### Mechanical tests for PCoPF_6_-Gel and PCoCl-Gel

The samples were measured using a Discovery HR-3, hybrid rheometer (TA Instrument) with a parallel-plate geometry. The hydrogel was cut into a circular disk with a thickness of 2 mm and a diameter of 25 mm. The gap between the two plates was accurately set by controlling the normal force. Oscillation amplitude measurement was performed to find the linear viscoelastic region. Frequency was 10.0 rad/s and strain was from 0.1% ~ 100.0%. Then, oscillatory frequency measurements were performed in the linear viscoelastic regime at 25 ^o^C. The strain was kept at 0.2% while the frequency was changed from 0.1 to 100 rad/s. The shear storage modulus (G’) and the shear loss modulus (G”) were then measured.

### Antimicrobial evaluation for PCoCl-Gel

The antimicrobial evaluation for PCoCl-Gel followed a procedure similar to those published in previous reports^23^,^55^,^56^. For these bacteria growing on agar plates, a single colony was inoculated in 5 mL Tryptic Soy broth (TSB) at 37 °C for 24 hours, shaking at 190 rpm/min. All bacteria were grown to an optical density of about l.00 (OD_600_ = ~1.00) for further use. A series of TSB medium solutions containing different concentrations of PCoCl-Gel (5, 10 and 20 mg/mL, dried weight) were prepared in test tubes. Then, 5 μL bacteria (O.D. = 1.00) was incubated in each test tube with a starting O.D. = ~0.05. The culture solution, without gels, was used as the control. The cultured solutions were incubated at 37 ^o^C. Bacterial growth was detected at OD_600_ and was compared to controls of TSB without gels. The percent inhibition was calculated, according Equation 4, which was determined by UV-vis at OD_600_. All assays were carried out in duplicate using the same assay plate.


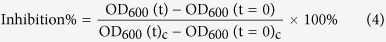


OD_600_ (t = 0) indicates the initial OD_600_ value, and OD_600_ (t) is the OD_600_ value for cells after incubation with gels for t hours. OD_600_ (t = 0)_c_ is the initial OD_600_ value and OD_600_ (t)_c_ is the OD_600_ value for control samples after incubation for t hours.

## Additional Information

**How to cite this article**: Zhang, J. *et al.* Anion-Responsive Metallopolymer Hydrogels for Healthcare Applications. *Sci. Rep.* **5**, 11914; doi: 10.1038/srep11914 (2015).

## Supplementary Material

Supplementary Information

## Figures and Tables

**Figure 1:**
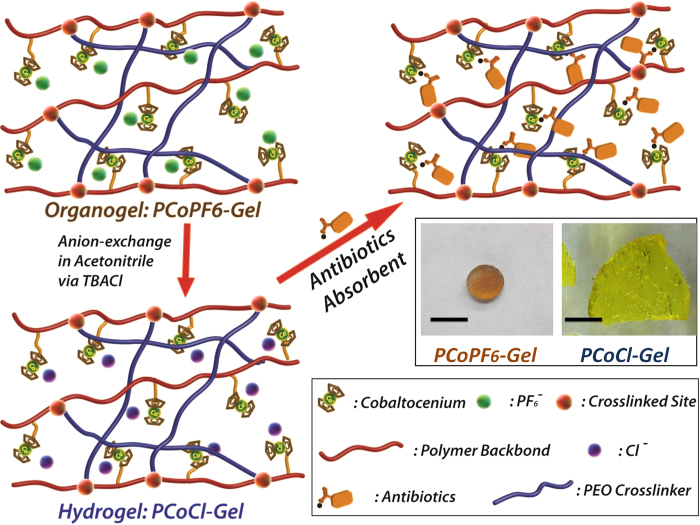
Illustration of anion-paired (Cl^−^ (purple circles), PF_6_− (green circles) and antibiotics) cobaltocenium-containing organogels and hydrogels. Inserted: optical images of two representative gels (Scale Bar: 1 cm).

**Figure 3:**
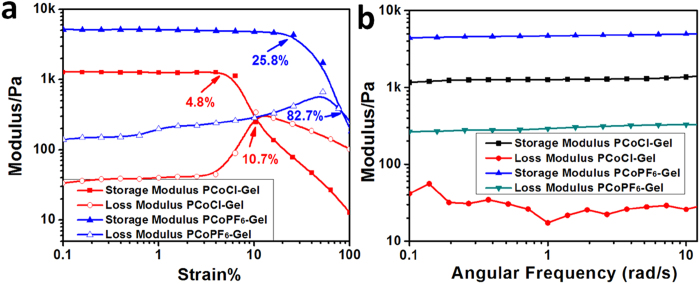
Mechanical properties of PCoCl-Gel; and PCoPF_6_-Gel: (**a**) The curves of shear storage modulus and shear loss modulus *vs.* strain for PCoCl-Gel and PCoPF_6_-Gel performed by amplitude sweep under the frequency of 10 rad/s. (**b**) The shear storage modulus and shear loss modulus with different frequency performed by a frequency sweep under the strain at 0.2%.
