# Supplementary material for: Anion-Responsive Metallopolymer Hydrogels for Healthcare Applications
Source: Sci Rep. 2015 Jul 23;5:11914. doi: 10.1038/srep11914 (PMC4511866; doi:10.1038/srep11914)
Supplement: Supplementary Information [file srep11914-s1.pdf]

Anion-Responsive Metallopolymer Hydrogels for Healthcare Applications

Jiuyang Zhang,† Jing Yan,† Parasmani Pageni,†Yi Yan,† Adam Wirth,† Yun-Ping Chen,‡ Yali, Qiao,† Qian Wang,† Alan W. Decho,‡ Chuanbing Tang†*

†Department of Chemistry and Biochemistry, and ‡Department of Environmental Health Sciences, University of South Carolina, Columbia, South Carolina 29208, USA

Supporting Information

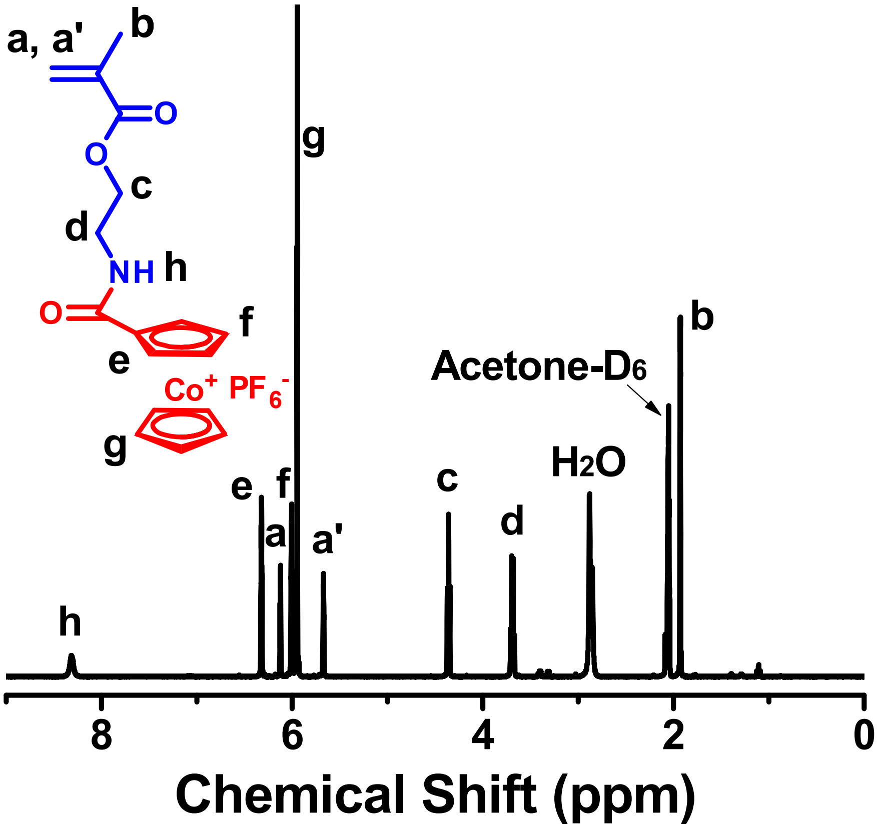

Figure S1. ^{1}H NMR spectrum of cobaltocenium-containing monomer ($CoAEMAPF_6$).

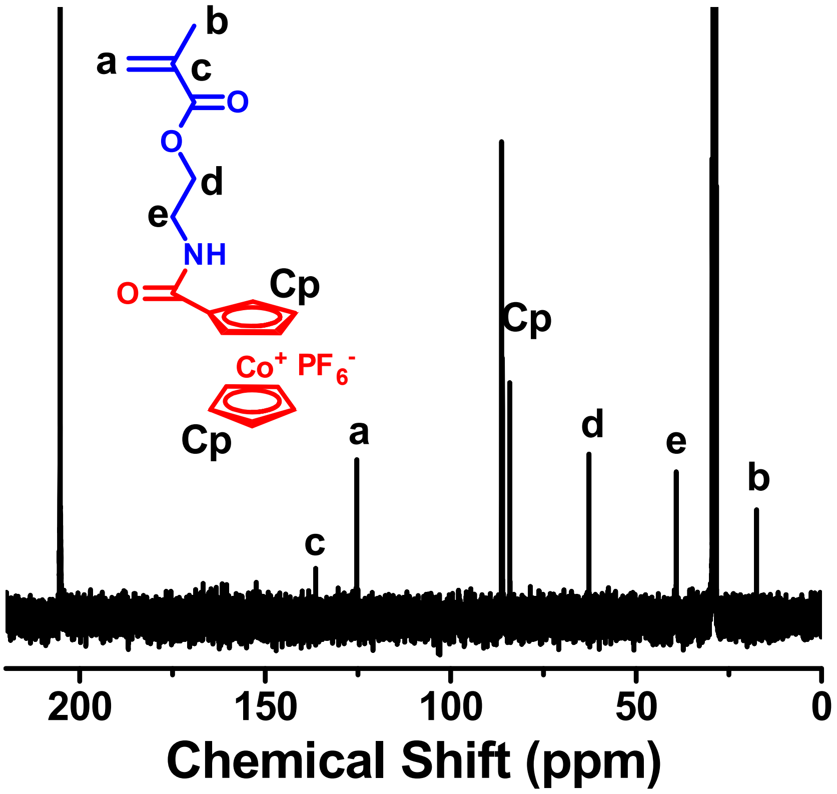

Figure S2. ^{13}C NMR spectrum of cobaltocenium-containing monomer ($CoAEMAPF_6$).

Scheme S1. Procedure for the measurement of compositions of cobaltocenium-containing gels ($PCoPF_6$-Gel).

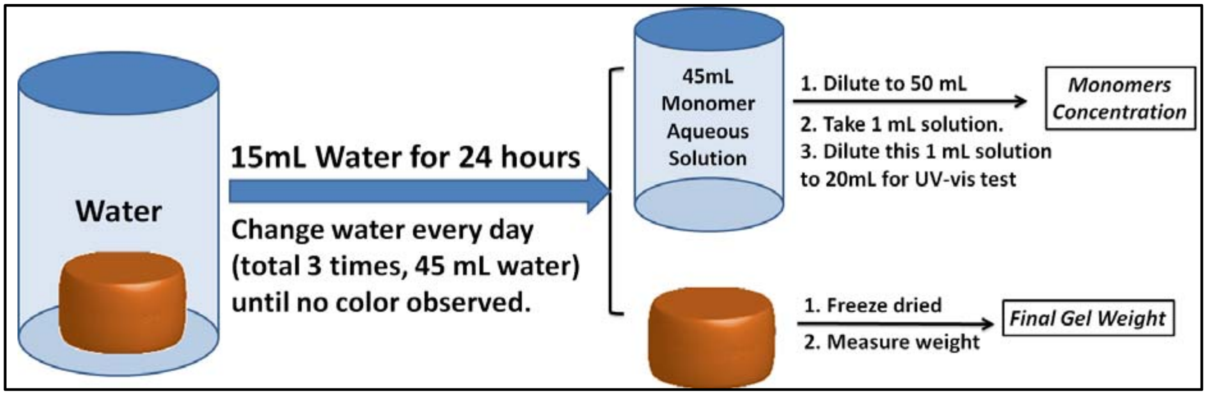

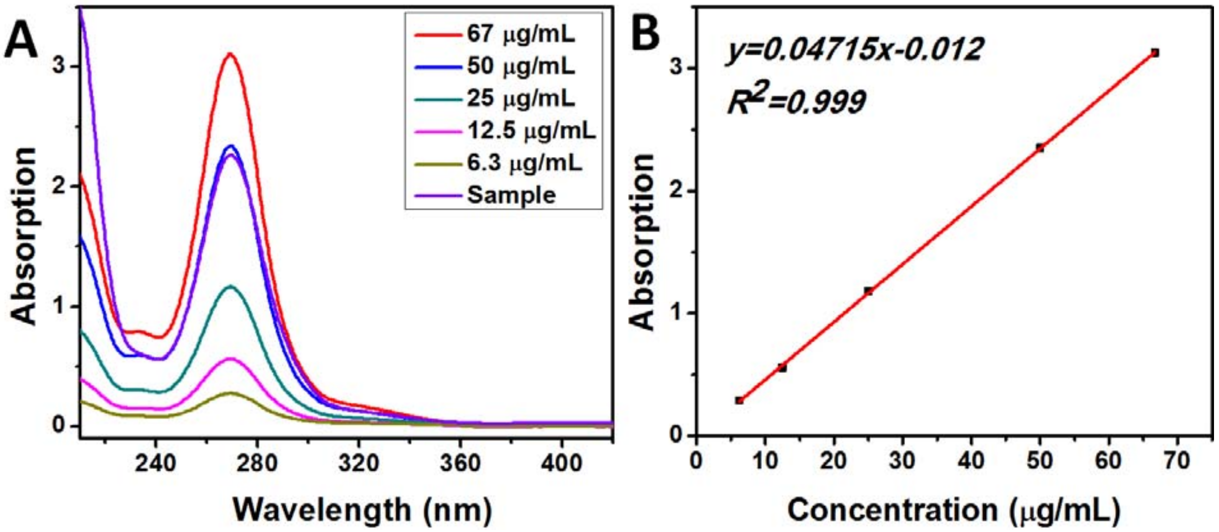

Figure S3. (A) UV-vis absorption of $CoAEMAPF_6$ in a series of solutions with different dilutions; (B) A standard curve of monomer concentrations from UV-vis absorption for the cobaltocenium-containing monomer ($CoAEMAPF_6$).

Scheme S2. A procedure for the preparation of a chloride-paired PCoCl-Gel hydrogel from a $PCoPF_6$-Gel organogel via tetrabutylammonium chloride (TBACl).

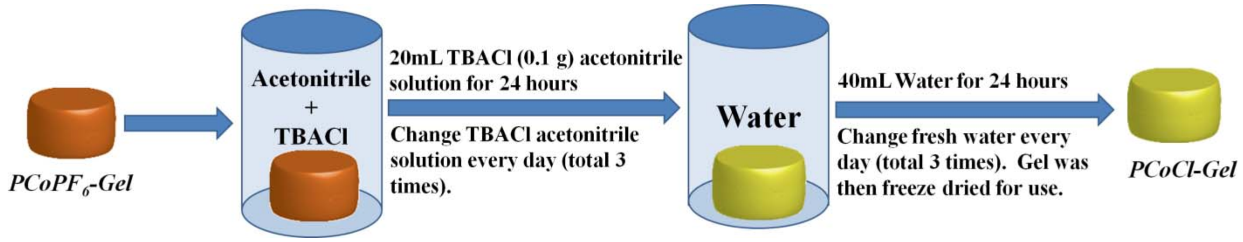

Table S1. Water contents of $PCoPF_6$-Gel and PCoCl-Gel.

$PCoPF_6$-Gel	Dry Gel (mg)	Wet Gel (mg)	Water Content%
1	18.7	87.8	78.7
2	26.1	129.0	79.8
3	26.5	148.2	82.1
Average	23.8	121.7	80.4
PCoCl-Gel	Dry Gel (mg)	Wet Gel (mg)	Water Content%
1	12.6	333.0	96.2
2	9.1	321.1	97.2
3	8.5	191.0	95.6
Average	10.1	281.7	96.4

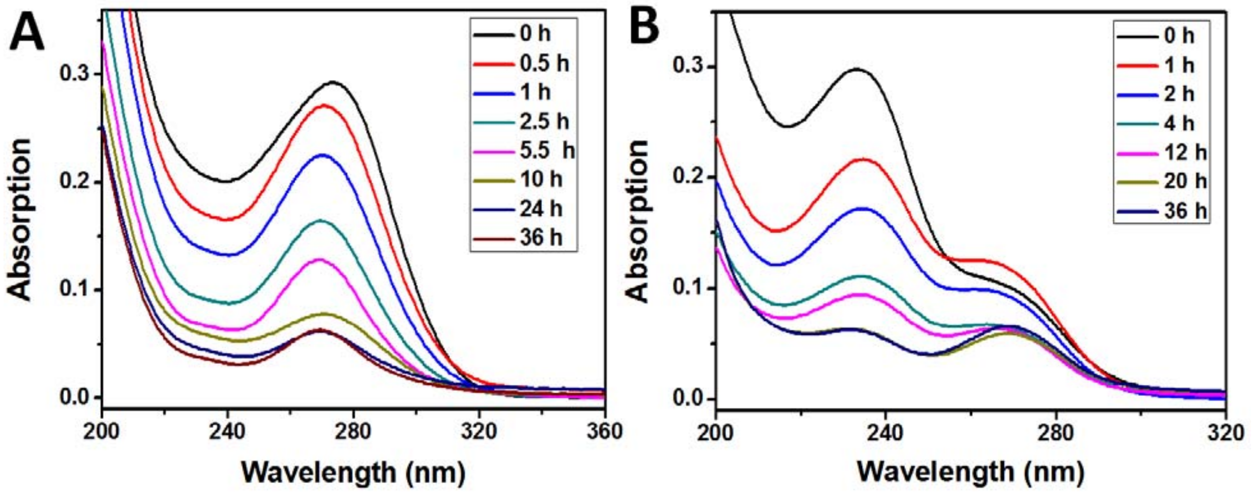

Figure S4. Time-dependent UV-vis absorption spectra using PCoCl-Gel (1.5 mg/mL) as the absorbent for (A) 10 mg/L cefazolin and (B) 10 mg/L cefoxitin.

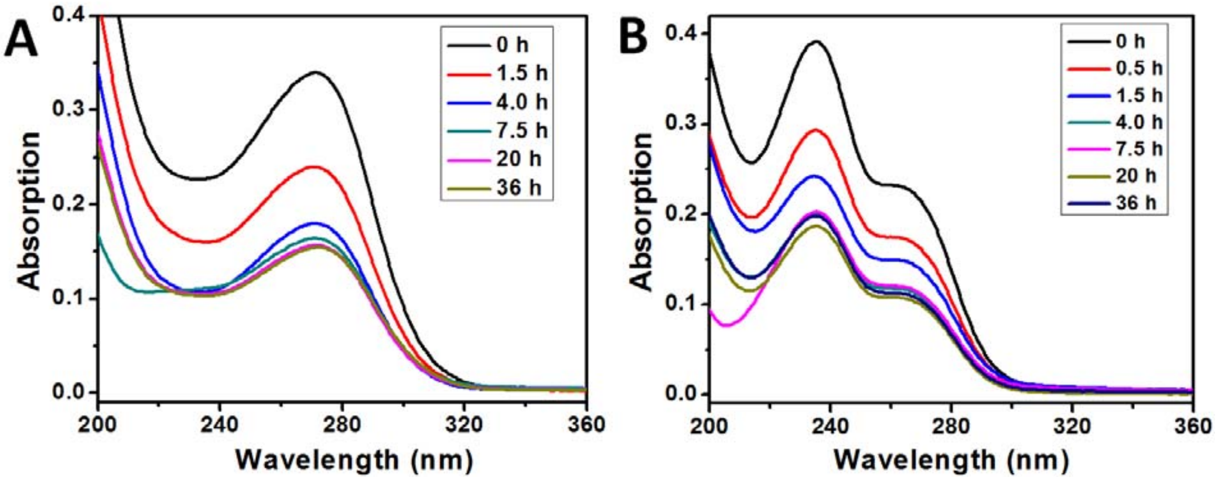

Figure S5. Time-dependent UV-vis absorption spectra using PCoCl-Gel (2.25 mg/mL) as the absorbent for (A) 2 g/L cefazolin and (B) 2 g/L cefoxitin solutions.

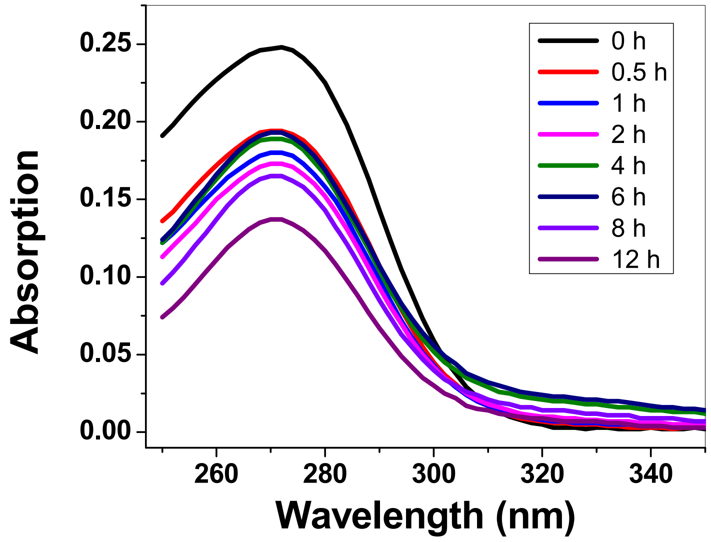

Figure S6. Time-dependent UV-vis absorption spectra using PCoCl-Gel (1.5 mg/mL) as the absorbent for 10 mg/L cefazolin in 3 mL tap water.

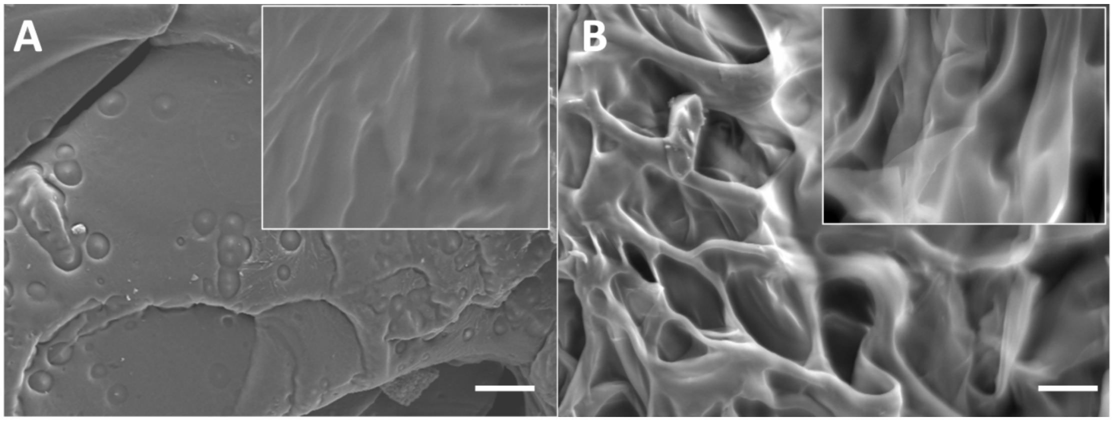

Figure S7. SEM images for (A) $PCoPF_6$-Gel and (B) PCoCl-Gel. Inserts are zoom-in pictures. Scale bars: 20 μm.

Theoretical calculation for the capacity of PCoCl-Gel to absorb antibiotics in water. Based on the composition for PF_6^- anion-paired gel (see the main text), the weight percentage of cobaltocenium moieties (***CoPF6%***) was 67.3%, which indicated 32.7% of the gel was PEO crosslinkers (***PEODMA%***). After fully exchanging PF_6^- anions to Cl^-, the moles for cobaltocenium moieties would not change. The weight percentage of chloride-paired cobaltocenium moieties (***CoCl%***) could be calculated according to Equation S1:

$$CoCl\% = \left(\frac{CoPF6\%}{M_{PF6}} \times M_{Cl}\right) / \left(\frac{CoPF6\%}{M_{PF6}} \times M_{Cl} + PEODMA\%\right) \quad \textbf{(Equation S1)}$$

$\boldsymbol{M_{PF6}}$ and $\boldsymbol{M_{Cl}}$ were molecular weights of PF_6^-- and chloride-paired cobaltocenium moieties, which are 488 g/mol and 378 g/mol, respectively. Therefore ***CoCl%*** would be **61.5%**.

Assuming that one cobaltocenium complexes with one antibiotic molecule, the amount of antibiotics taken by 1 mg PCoCl-Gel can be calculated by following Equation S2:

$$m = \frac{1\ mg \times CoCl\%}{M_{Cl}} \times M_{Antibiotics}$$ **(Equation S2)**

$\boldsymbol{M_{Antibiotics}}$ is the molecular weight of antibiotics. For example, for amoxicillin, the molecular weight is 364 g/mol. Then, **m** would be 0.60 mg, which indicates that the **PCoCl-Gel** can take up to 0.60 mg amoxicillin in aqueous condition.
